# Non-Gaussian Closed Form Solutions for Geometric Average Asian Options in the Framework of Non-Extensive Statistical Mechanics

**DOI:** 10.3390/e20010071

**Published:** 2018-01-18

**Authors:** Pan Zhao, Benda Zhou, Jixia Wang

**Affiliations:** 1 College of Finance and Mathematics, West Anhui University, Lu’an 237012, China; 2 Financial Risk Intelligent Control and Prevention Institute of West Anhui University, Lu’an 237012, China; 3 School of Mathematics and Information Sciences, Henan Normal University, Xinxiang 453002, China

**Keywords:** geometric average Asian option, Non-extensive statistics, martingale method

## Abstract

In this paper we consider pricing problems of the geometric average Asian options under a non-Gaussian model, in which the underlying stock price is driven by a process based on non-extensive statistical mechanics. The model can describe the peak and fat tail characteristics of returns. Thus, the description of underlying asset price and the pricing of options are more accurate. Moreover, using the martingale method, we obtain closed form solutions for geometric average Asian options. Furthermore, the numerical analysis shows that the model can avoid underestimating risks relative to the Black-Scholes model.

## 1. Introduction

It is very important for investors to accurately describe the law of asset price movement, which is the foundation of the pricing and risk management of derivatives. We know that the stock price model based on Brownian motion has appeared in many literatures. For example, Black and Scholes [1] and Merton [2] studied the pricing of financial derivatives of the underlying stock following such a model. It is well known that the hypothesis that the asset price changes follow Brownian motion implies that the price changes are independent and the distributions of log-returns are normal, but a number of studies have shown that empirical distributions of returns are not normal distributions. Researchers have found that long-term dependence of prices is common in stock markets all over the world [3,4,5,6] and the distributions of returns usually exhibit fat tails [7,8,9].

Recently, along with the development of Tsallis non-extensive thermostatistics, some studies have suggested that the power-law distributions characteristic of the Tsallis non-extensive statistics framework can well model the distributions of the returns of some financial asset. For example, Rak and Drożdż [10] studied the Polish stock market and found that the Tsallis distribution of index *q* provides a satisfactory representation of the distribution of returns. Queirós and Moyano [11], and Biró and Rosenfeld [12] found that the empirical distribution of returns of the Dow Jones Industrial Average can be well fitted by a Tsallis distribution. Kozaki and Sato [13] found that the distribution of the S&P100 index returns is well described by a Tsallis distribution. Ryuji and Masayoshi [14] found that the time-series of foreign exchange rates can be well fitted by the Tsallis non-extensive statistics. Yuri and Katz [15] studied leverage returns and default risk valuations using Tsallis distributions. In addition, Borland [16,17] proposed a stock price model within the non-extensive statistical mechanics framework, in which the driving noise followed a statistical feedback process that can describe fat-tail characteristics of distributions of stock returns. Using the model, Borland derived closed form solutions for European options. Furthermore, empirical results showed that the option pricing model can closely reproduce market prices.

An Asian option is a special type of option contract, which is a path-depending exotic option whose terminal payoff depends on the average underlying price over some pre-set period of time [18,19,20,21,22]. Hence it has a lower volatility than its underlying asset and is less subject to price manipulation. Asian options are broadly segregated into two categories: geometric average Asian options and arithmetic average Asian options. Due to their popularity, Asian options have been studied by a number of researchers. For example, Caverhill and Clewlow [23] obtained numerical approximations of the price of Asian options using the fast Fourier transform. Alziary et al. [24] obtained approximate formulas for the Asian options using the partial differential equation method. Devreesea et al. [25] derived closed form solutions for the price of geometric average Asian options by the use of the path integral approach. Wang et al. [26] obtained closed form solutions for geometric average Asian options using the Lie group analysis method. But in the above literatures, underlying stocks were driven by the geometric Brownian motion. In the first paragraph, we have introduced that the return curve of underlying stock usually has a peak and fat tail phenomenon in real financial markets, not is a normal distribution. That is to say, the use of geometric Brownian motion to describe the underlying stock price is inaccurate. This usually makes the price of options higher than the actual value and causes investors to underestimate the risk. Therefore, in order to more accurately describe the movement of underlying stock price, several researchers have tried to replace the geometric Brownian motion with others recently. For example, Chung et al. [27] employed a mean reversion and jump process to describe the movement of underlying stock price instead of the geometric Brownian motion and studied the pricing of arithmetic average Asian options. Chiu et al. [28] studied the pricing of Asian options under Lévy processes and obtained efficiently approximate formulas by the Fast Fourier Transform.

In this paper, in order to describe the movement of underlying stock price more accurately, we consider an asset price model with a Tsallis distribution of index q in the framework of non-extensive statistical mechanics. The model can describe the peak and fat tail characteristics of returns. Moreover, we study the pricing of geometric average Asian options and obtain closed form solutions by the use of the martingale method.

The paper is organized as follows. In Section 2, we model a price process of an underlying stock using Tsallis non-extensive statistical mechanics. In Section 3, we derive the analytic formulas and call-put parity relationship of geometric average Asian options. In Section 4, we carry out a comparative analysis between the model and the Black-Scholes model based on the geometric Brown motion. In the final section, we summarize the paper.

## 2. Market Model

Suppose that there are two types of tradable assets in a continuous financial market. One is a risk-free asset, called a bond. Its price *B*(*t*) follows the equation

(1) $$\left\{\begin{array}{c} dB(t)=rB(t)dt\\ B(0)=1 \end{array}\right.$$

where *r* is a risk-free interest rate. The other one is a risky asset, called a stock. Its price *S*(*t*) satisfies the stochastic differential equation

(2) $$\left\{\begin{array}{c} dS(t)=\mu S(t)dt+\sigma S(t)d\Omega (t)\\ S\left(0\right)=S_{0} \end{array}\right.$$

where

$$d\Omega \left(t\right)=P{\left(\Omega \left(t\right)\right)}^{\frac{1-q}{2}}dW\left(t\right)$$

(3) $$P\left(\Omega \left(t\right),t\right)=\frac{1}{z(t)}{\left(1-\beta \left(t\right)\left(1-q\right)\Omega {\left(t\right)}^{2}\right)}^{\frac{1}{1-q}}$$

(4) $$z\left(t\right)=\int_{-\infty }^{+\infty }{\left(1-\left(1-q\right)\beta \left(t\right)\Omega {\left(t\right)}^{2}\right)}^{\frac{1}{1-q}}d\Omega \left(t\right)={\left(\left(2-q\right)\left(3-q\right)ct\right)}^{\frac{1}{3-q}}$$

(5) $$\beta \left(t\right)=c^{\frac{1-q}{3-q}}{\left(\left(2-q\right)\left(3-q\right)t\right)}^{\frac{2}{q-3}}$$

$1<q<\frac{5}{3},c=\frac{\pi }{q-1}\frac{\Gamma^{2}\left(\frac{1}{q-1}-\frac{1}{2}\right)}{\Gamma^{2}\left(\frac{1}{q-1}\right)},\Gamma \left(\cdot \right)$ is a Gamma function. Let (Ω,F,P) be a complete probability space. W(t) is a standard Brownian motion on the probability space (Ω,F,P). The probability density function (3) is called Tsallis distribution [16]. Its mean is zero and variance is

(6) $$Var\left[\Omega \left(t\right)\right]=\frac{1}{\left(5-3q)\beta (t\right)}$$

Furthermore, the probability density function (3) satisfies nonlinear Fokker-Planck equation

(7) $$\frac{\partial P(\Omega (t),t)}{\partial t}=\frac{1}{2}\frac{\partial P^{2-q}\left(\Omega \left(t\right),t\right)}{\partial \Omega {\left(t\right)}^{2}}$$

It is not difficult to find that when q>1 the distribution exhibits fat tails which can describe long-term memory of asset price, when q→1 it reduces to Gaussian distribution and hence the standard model is recovered. In order to price geometric average Asian options, we first introduce the following three lemmas.

**Lemma** **1.** *For any given time t and T, between the random variables Ω(t) and Ω(T) there exists a transformation*

(8) $$\Omega \left(t\right)=\sqrt{\frac{\beta (T)}{\beta (t)}}\Omega \left(T\right)$$

**Proof** **of** **Lemma** **1.** For any given time *t*, the probability density distribution (3) can be mapped onto the distribution of the standardized random variable $x^{*}$ through the transformation

(9) $$x^{*}=\sqrt{\frac{\beta (t)}{\beta^{*}}}\Omega \left(t\right)$$

with the probability density distribution

(10) $$P\left(x^{*}\right)=\frac{1}{z^{*}}{\left(1-\beta^{*}\left(1-q\right){x^{*}}^{2}\right)}^{\frac{1}{1-q}}$$

Substituting (9) into (4), we have

(11) $$\begin{array}{ccc} z^{*} & = & \int_{-\infty }^{+\infty }{\left(1-\left(1-q\right)\beta^{*}{x^{*}}^{2}\right)}^{\frac{1}{1-q}}dx^{*}\\ & = & \sqrt{\frac{\beta (t)}{\beta^{*}}}\int_{-\infty }^{+\infty }{\left(1-\left(1-q\right)\beta \left(t\right)\Omega {\left(t\right)}^{2}\right)}^{\frac{1}{1-q}}d\Omega \left(t\right)\\ & = & \sqrt{\frac{\beta (t)}{\beta^{*}}}z\left(t\right) \end{array}$$

Substituting (9) and (11) into (10), thus, we obtain the relation

$$\begin{array}{ccc} P(x^{*}) & = & \sqrt{\frac{\beta^{*}}{\beta (t)}}\frac{1}{z}{\left(1-\beta \left(t\right)\left(1-q\right)\Omega {\left(t\right)}^{2}\right)}^{\frac{1}{1-q}}\\ & = & \sqrt{\frac{\beta^{*}}{\beta (t)}}P\left(\Omega \left(t\right),t\right) \end{array}$$

For a given time *T*, we can also map the standardized distribution of $x^{*}$ onto the distribution of Ω(t) through transformation

(12) $$\Omega \left(t\right)=\sqrt{\frac{\beta^{*}}{\beta (T)}}x^{*}$$

From (9) and (12), we have that for any given time *t* and *T*, between the random variables Ω(t) and Ω(T) there exists a transformation

$$\Omega \left(t\right)=\sqrt{\frac{\beta (T)}{\beta (t)}}\Omega \left(T\right)$$

 ◻

Let

(13) $$\gamma \left(t\right)=\frac{\mu -r}{\sigma P_{q}^{\frac{1-q}{2}}}$$

(14) $$\tilde{W}\left(t\right)=W\left(t\right)+\int_{0}^{t}\gamma \left(s\right)ds$$

and $E[\exp\left(\frac{1}{2}\int_{0}^{t}\gamma^{2}\left(s\right)ds\right)]<\infty$ [29]. Then, we can define an equivalent martingale measure Q, which is related to P through the Radon-Nikodym derivative

(15) $$\frac{dQ}{dP}=\exp\left(\int_{0}^{t}\gamma \left(s\right)dW\left(s\right)-\frac{1}{2}\int_{0}^{t}\gamma^{2}\left(s\right)ds\right)$$

According to the Girsanov theorem, we know that $\tilde{W}\left(t\right)$ is the standard Brownian motion under the probability measure Q.

**Lemma** **2.** Under the probability measure Q, the discounted stock price process $S^{*}\left(t\right)=e^{-rt}S\left(t\right)$ is a martingale.

**Proof** **of** **Lemma** **2.** Substituting (13) and (14) into the first equation of (2), we have

(16) $$\begin{array}{ccc} dS(t) & = & \mu S(t)dt+\sigma S(t)d\Omega (t)\\ & = & \mu S\left(t\right)dt+\sigma S\left(t\right)P{\left(\Omega \left(t\right)\right)}^{\frac{1-q}{2}}dW\left(t\right)\\ & = & rS\left(t\right)dt+\sigma S\left(t\right)P{\left(\Omega \left(t\right)\right)}^{\frac{1-q}{2}}d\tilde{W}\left(t\right) \end{array}$$

Applying the Itô formula to the discounted process $S^{*}\left(t\right)=e^{-rt}S\left(t\right)$, we obtain

$$\begin{array}{ccc} dS^{*}\left(t\right) & = & \left(-re^{-rt}S\left(t\right)+e^{-rt}S\left(t\right)r\right)dt+e^{-rt}S\left(t\right)\sigma P_{q}^{\frac{1-q}{2}}d\tilde{W}\left(t\right)\\ & = & S^{*}\left(t\right)\sigma P_{q}^{\frac{1-q}{2}}d\tilde{W}\left(t\right) \end{array}$$

Since $\tilde{W}\left(t\right)$ is the standard Brownian motion under the probability measure Q, the discounted process $S^{*}\left(t\right)$ is a martingale. ◻

**Lemma** **3.** *Under the probability measure Q, the stochastic differential Equation (2) has a solution*

(17) $$S\left(t\right)=S\left(0\right)\exp\{rt-\frac{1}{2}\sigma^{2}at^{\frac{2}{3-q}}+\frac{1}{2}\sigma^{2}ab\left(1-q\right)\Omega {\left(t\right)}^{2}+\sigma \Omega \left(t\right)\}$$

*where $a=\frac{1}{2}{\left(\left(2-q\right)\left(3-q\right)c\right)}^{\frac{q-1}{3-q}}$, $b=c^{\frac{1-q}{3-q}}{\left(\left(2-q\right)\left(3-q\right)\right)}^{-\frac{2}{3-q}}$.*

**Proof** **of** **Lemma** **3.** Applying the Itô formula to lnS(t) and substituting (16), we get

(18) $$\begin{array}{ccc} d\ln S(t) & = & (rS\left(t\right)\frac{\partial \ln S(t)}{\partial S(t)}+\frac{1}{2}\sigma^{2}P^{1-q}S{\left(t\right)}^{2}\frac{\partial^{2}\ln S\left(t\right)}{\partial S{\left(t\right)}^{2}})dt\\ & & +\;\sigma P^{\frac{1-q}{2}}S\left(t\right)\frac{\partial \ln S(t)}{\partial S(t)}d\tilde{W}\left(t\right)\\ & = & \left(r-\frac{1}{2}\sigma^{2}P^{1-q}\right)dt+\sigma P^{\frac{1-q}{2}}d\tilde{W}\left(t\right) \end{array}$$

Over the time interval [0,t], taking the integral of (18), we have

(19) $$\ln S\left(t\right)-\ln S\left(0\right)=rt-\frac{1}{2}\sigma^{2}\int_{0}^{t}P{\left(\Omega \left(v\right)\right)}^{1-q}dv+\sigma \int_{0}^{t}P{\left(\Omega \left(v\right)\right)}^{\frac{1-q}{2}}d\tilde{W}\left(t\right)$$

Taking the exponent of (19), we obtain

$$\begin{array}{ccc} S(t) & = & S\left(0\right)\exp\{rt-\frac{1}{2}\sigma^{2}\int_{0}^{t}P^{1-q}dv+\sigma \int_{0}^{t}P^{\frac{1-q}{2}}d\tilde{W}\left(v\right)\}\\ & = & S\left(0\right)\exp\{rt-\frac{1}{2}\sigma^{2}\int_{0}^{t}\left[z{\left(v\right)}^{q-1}\left(1-\beta \left(v\right)\left(1-q\right)\Omega {\left(v\right)}^{2}\right)\right]dv+\sigma \Omega \left(t\right)\}\\ & = & S\left(0\right)\exp\{rt-\frac{1}{2}\sigma^{2}\int_{0}^{t}\left[{\left(\left(2-q\right)\left(3-q\right)cv\right)}^{\frac{q-1}{3-q}}\left(1-\beta \left(v\right)\left(1-q\right)\Omega {\left(v\right)}^{2}\right)\right]dv\\ & & +\sigma \Omega (t)\} \end{array}$$

Using Lemma 1, we have the above equation

$$=S\left(0\right)\exp\{rt-\frac{1}{2}\sigma^{2}\int_{0}^{t}\left[{\left(\left(2-q\right)\left(3-q\right)cv\right)}^{\frac{q-1}{3-q}}\left(1-\beta \left(t\right)\left(1-q\right)\Omega {\left(t\right)}^{2}\right)\right]dv+\sigma \Omega \left(t\right)\}$$

Let $a=\frac{1}{2}{\left(\left(2-q\right)\left(3-q\right)c\right)}^{\frac{q-1}{3-q}}$, $b=c^{\frac{1-q}{3-q}}{\left(\left(2-q\right)\left(3-q\right)\right)}^{-\frac{2}{3-q}}$, we get the above equation

$$\begin{array}{c} =S\left(0\right)\exp\{rt-\frac{1}{2}\sigma^{2}at^{\frac{2}{3-q}}\left(1-b\left(1-q\right)t^{-\frac{2}{3-q}}\Omega {\left(t\right)}^{2}\right)+\sigma \Omega \left(t\right)\}\\ =S\left(0\right)\exp\{rt-\frac{1}{2}\sigma^{2}at^{\frac{2}{3-q}}+\frac{1}{2}\sigma^{2}ab\left(1-q\right)\Omega {\left(t\right)}^{2}+\sigma \Omega \left(t\right)\} \end{array}$$

◻

In the above calculation process, we equate distribution equality to almost sure equality. Therefore, there is a little regret that there may be arbitrages.

## 3. Pricing Geometric Average Asian Options

Assume that there are geometric average Asian options with the strike price *K* and expiration date *T*. Let $G_{T}=\exp\left\{\frac{1}{T}\int_{0}^{T}\ln S\left(t\right)dt\right\}$. In the risk-neutral framework, the value of an option is equal to the expected value of discounted price at the risk-free rate under the equivalent martingale measure Q. Thus, the value of a geometric average Asian call option can be written as

(20) $$C_{q}\left(K,T\right)=E_{Q}\left[e^{-rT}{\left(G_{T}-K\right)}^{+}\right]$$

The value of a geometric average Asian put option can be written as

(21) $$P_{q}\left(K,T\right)=E_{Q}\left[e^{-rT}{\left(K-G_{T}\right)}^{+}\right]$$

where ${\left(x\right)}^{+}$ is x>0. We will give pricing formulas of geometric average Asian call and put options in the following theorems.

**Theorem** **1.** *Suppose there is a geometric average Asian call option with the strike price K and expiration date T, and the underlying stock price follows the stochastic differential Equation (2). Then, under the risk neutral framework its value can be written as*

(22) $$\begin{array}{c} C_{q}(K,T)=\frac{S(0)}{z(T)}e^{-\frac{1}{2}rT-\frac{1}{2}\frac{3-q}{5-q}\sigma^{2}aT^{\frac{2}{3-q}}}\int_{r_{1}}^{r_{2}}\exp\left\{\frac{1}{2}\sigma^{2}ab\left(1-q\right)x^{2}+\sigma x\right\}\;\times \\ \;(1-\left(1\;\;-\;\;q\right)\beta \left(T\right)x^{2}{)}^{\frac{1}{1-q}}dx\;-\;\frac{K}{z(t)}e^{-rT}\;\int_{r_{1}}^{r_{2}}{\left(1-\left(1-q\right)\beta \left(T\right)x^{2}\right)}^{\frac{1}{1-q}}dx \end{array}$$

*where*

$$\begin{array}{ccc} r_{1} & = & \frac{-1-{\left[1-2ab\left(1-q\right)\left(\frac{1}{2}rT-\frac{1}{2}\frac{3-q}{5-q}\sigma^{2}aT^{\frac{2}{3-q}}+\ln S\left(0\right)-\ln K\right)\right]}^{\frac{1}{2}}}{\sigma ab(1-q)}\\ r_{2} & = & \frac{-1+{\left[1-2ab\left(1-q\right)\left(\frac{1}{2}rT-\frac{1}{2}\frac{3-q}{5-q}\sigma^{2}aT^{\frac{2}{3-q}}+\ln S\left(0\right)-\ln K\right)\right]}^{\frac{1}{2}}}{\sigma ab(1-q)}\\ a & = & \frac{1}{2}{\left(\left(2-q\right)\left(3-q\right)c\right)}^{\frac{q-1}{3-q}},b=c^{\frac{1-q}{3-q}}{\left(\left(2-q\right)\left(3-q\right)\right)}^{-\frac{2}{3-q}} \end{array}$$

**Proof** **of** **Theorem** **1.** Calculating (20), we have

$$\begin{array}{ccc} C_{q}\left(K,T\right) & = & E_{Q}{\left[e^{-rT}\left(G_{T}-K\right)\right]}_{\left\{G_{T}>K\right\}}\\ & = & E_{Q}{\left[e^{-rT}G_{T}\right]}_{\left\{G_{T}>K\right\}}-E_{Q}{\left[e^{-rT}K\right]}_{\left\{G_{T}>K\right\}}\\ & ≜ & I-⨿ \end{array}$$

where

$$\begin{array}{ccc} I & = & E_{Q}{\left[e^{-rT}G_{T}\right]}_{\left\{G_{T}>K\right\}} \end{array}$$

$$\begin{array}{ccc} ⨿ & = & E_{Q}{\left[e^{-rT}K\right]}_{\left\{G_{T}>K\right\}} \end{array}$$

In order to calculate I and ⨿, we first solve the inequality $\left\{G_{T}>K\right\}$. Substituting $G_{T}=\exp\left\{\frac{1}{T}\int_{0}^{T}\ln S\left(t\right)dt\right\}$, we obtain

(23) $$G_{T}>K⟺\exp\left\{\frac{1}{T}\int_{0}^{T}\ln S\left(t\right)dt\right\}>K$$

Taking the log of (23), we have

$$\begin{array}{c} \frac{1}{T}\int_{0}^{T}\ln S\left(t\right)dt>\ln K\\ \int_{0}^{T}\ln S\left(t\right)dt>T\ln K \end{array}$$

Substituting (17), we get

$$\int_{0}^{T}\ln\left(S\left(0\right)\exp\left\{rt-\frac{1}{2}\sigma^{2}at^{\frac{2}{3-q}}+\frac{1}{2}\sigma^{2}ab\left(1-q\right)x^{2}+\sigma x\right\}\right)dt>T\ln K$$

Calculating the log

$$\int_{0}^{T}\left(\ln S\left(0\right)+rt-\frac{1}{2}\sigma^{2}at^{\frac{2}{3-q}}+\frac{1}{2}\sigma^{2}ab\left(1-q\right)x^{2}+\sigma x\right)dt>T\ln K$$

Calculating the integral

$$T\ln S\left(0\right)+\frac{1}{2}rT^{2}-\frac{1}{2}\frac{3-q}{5-q}\sigma^{2}aT^{\frac{5-q}{3-q}}+\frac{1}{2}\sigma^{2}ab\left(1-q\right)Tx^{2}+\sigma Tx>T\ln K$$

Multiplying the $\frac{1}{T}$

$$\ln S\left(0\right)+\frac{1}{2}rT-\frac{1}{2}\frac{3-q}{5-q}\sigma^{2}aT^{\frac{2}{3-q}}+\frac{1}{2}\sigma^{2}ab\left(1-q\right)x^{2}+\sigma x>\ln K$$

Transposing

(24) $$\frac{1}{2}\sigma^{2}ab\left(1-q\right)x^{2}+\sigma x+\frac{1}{2}rT-\frac{1}{2}\frac{3-q}{5-q}\sigma^{2}aT^{\frac{2}{3-q}}+\ln S\left(0\right)-\ln K>0$$

Now, we need to solve the quadratic inequality (24). Owing to $1<q<\frac{5}{3}$, it is not difficult for us to know a>0,b>0. Thus, the coefficient $\frac{1}{2}\sigma^{2}ab\left(1-q\right)<0$. In addition, let

$$K<\exp\{\frac{1}{2}rT+\ln S\left(0\right)-\frac{1}{2}\frac{3-q}{5-q}\sigma^{2}aT^{\frac{2}{3-q}}-\frac{1}{2ab(1-q)}\}$$

Then, the discriminant

$$▵=\sigma^{2}-4\times \frac{1}{2}\sigma^{2}ab\left(1-q\right)\left(\frac{1}{2}rT-\frac{1}{2}\frac{3-q}{5-q}\sigma^{2}aT^{\frac{2}{3-q}}+\ln S\left(0\right)-\ln K\right)>0$$

Hence the quadratic equation

(25) $$\frac{1}{2}\sigma^{2}ab\left(1-q\right)x^{2}+\sigma x+\frac{1}{2}rT-\frac{1}{2}\frac{3-q}{5-q}\sigma^{2}aT^{\frac{2}{3-q}}+\ln S\left(0\right)-\ln K=0$$

has two roots

$$\begin{array}{ccc} r_{1,2} & = & \frac{-\sigma \pm {\left[\sigma^{2}-2ab\sigma^{2}\left(1-q\right)\left(\frac{1}{2}rT-\frac{1}{2}\frac{3-q}{5-q}\sigma^{2}aT^{\frac{2}{3-q}}+\ln S\left(0\right)-\ln K\right)\right]}^{\frac{1}{2}}}{\sigma^{2}ab\left(1-q\right)}\\ & = & \frac{-1\pm {\left[1-2ab\left(1-q\right)\left(\frac{1}{2}rT-\frac{1}{2}\frac{3-q}{5-q}\sigma^{2}aT^{\frac{2}{3-q}}+\ln S\left(0\right)-\ln K\right)\right]}^{\frac{1}{2}}}{\sigma ab(1-q)} \end{array}$$

So, the solutions of the inequality $\left\{G_{T}>K\right\}$ are $x\in (r_{1},r_{2})$.Using this, then we can calculate I and ⨿. Substituting the roots into I, we have

(26) $$\begin{array}{ccc} I & = & \int_{r_{1}}^{r_{2}}e^{-rT}\exp\left\{\frac{1}{T}\int_{0}^{T}\ln S\left(t\right)dt\right\}P\left(x,T\right)dx\\ & = & \int_{r_{1}}^{r_{2}}e^{-rT}\exp\{\ln S\left(0\right)+\frac{1}{2}rT-\frac{1}{2}\frac{3-q}{5-q}\sigma^{2}aT^{\frac{2}{3-q}}+\frac{1}{2}\sigma^{2}ab\left(1-q\right)x^{2}\;+\\ & & \sigma x\}\times \frac{1}{z(T)}{\left(1-\left(1-q\right)\beta \left(T\right)x^{2}\right)}^{\frac{1}{1-q}}dx\\ & = & \frac{S(0)}{z(T)}e^{\frac{1}{2}rT-\frac{1}{2}\frac{3-q}{5-q}\sigma^{2}aT^{\frac{2}{3-q}}}\int_{r_{1}}^{r_{2}}\exp\left\{\frac{1}{2}\sigma^{2}ab\left(1-q\right)x^{2}+\sigma x\right\}\times \\ & & {\left(1-\left(1-q\right)\beta \left(T\right)x^{2}\right)}^{\frac{1}{1-q}}dx \end{array}$$

Substituting the roots into ⨿, we get

(27) $$\begin{array}{ccc} ⨿ & = & \int_{r_{1}}^{r_{2}}e^{-rT}KP\left(x,T\right)dx\\ & = & \frac{K}{z(T)}e^{-rT}\int_{r_{1}}^{r_{2}}{\left(1-\left(1-q\right)\beta \left(T\right)x^{2}\right)}^{\frac{1}{1-q}}dx \end{array}$$

Subtracting (27) from (26), we can obtain the value of a geometric average Asian call option is

$$\begin{array}{cc} C_{q}\left(K,T\right)= & \frac{S(0)}{z(T)}e^{-\frac{1}{2}rT-\frac{1}{2}\frac{3-q}{5-q}\sigma^{2}aT^{\frac{2}{3-q}}}\int_{r_{1}}^{r_{2}}\exp\left\{\frac{1}{2}\sigma^{2}ab\left(1-q\right)x^{2}+\sigma x\right\}\times \\ & {\left(1\;-\;\left(1\;-\;q\right)\beta \left(T\right)x^{2}\right)}^{\frac{1}{1-q}}dx\;-\;\frac{K}{z(t)}e^{-rT}\;\int_{r_{1}}^{r_{2}}{\left(1\;-\;\left(1\;-\;q\right)\beta \left(T\right)x^{2}\right)}^{\frac{1}{1-q}}dx \end{array}$$

◻

**Theorem** **2.** *Suppose there is a geometric average Asian put option with the strike price K and expiration date T, and the underlying stock price follows the stochastic differential Equation (2). Then, under the risk neutral framework its value can been written as*

(28) $$\begin{array}{cc} P_{q}\left(K,T\right)= & \frac{K}{z(T)}e^{-rT}\left[1-\int_{r_{1}}^{r_{2}}{\left(1-\left(1-q\right)\beta \left(T\right)x^{2}\right)}^{\frac{1}{1-q}}dx\right]\\ & -\;\frac{S(0)}{z(T)}e^{-\frac{1}{2}rT-\frac{1}{2}\frac{3-q}{5-q}\sigma^{2}aT^{\frac{2}{3-q}}}[\int_{-\infty }^{r_{1}}\exp\left\{\frac{1}{2}\sigma^{2}ab\left(1-q\right)x^{2}+\sigma x\right\}\;\times \\ & {\left(1-\left(1-q\right)\beta \left(T\right)x^{2}\right)}^{\frac{1}{1-q}}dx+\int_{r_{2}}^{+\infty }\exp\left\{\frac{1}{2}\sigma^{2}ab\left(1-q\right)x^{2}+\sigma x\right\}\;\times \\ & {\left(1-\left(1-q\right)\beta \left(T\right)x^{2}\right)}^{\frac{1}{1-q}}dx] \end{array}$$

**Proof** **of** **Theorem** **2.** Similar to the proof of theorem 1, we first solve the inequality $\left\{G_{T}>K\right\}$ and easily get the solutions $x\in \left(-\infty ,r_{1}\right)\cup \left(r_{2},+\infty \right)$. Calculating (21), we have

$$\begin{array}{ccc} P_{q}\left(K,T\right) & = & E_{Q}{\left[e^{-rT}K\right]}_{\left\{K>G_{T}\right\}}-E_{Q}{\left[e^{-rT}G_{T}\right]}_{\left\{K>G_{T}\right\}}\\ & = & \frac{K}{z(T)}e^{-rT}\left[1-\int_{r_{1}}^{r_{2}}{\left(1-\left(1-q\right)\beta \left(T\right)x^{2}\right)}^{\frac{1}{1-q}}dx\right]\\ & & -\;\frac{S(0)}{z(T)}e^{-\frac{1}{2}rT-\frac{1}{2}\frac{3-q}{5-q}\sigma^{2}aT^{\frac{2}{3-q}}}[\int_{-\infty }^{r_{1}}\exp\left\{\frac{1}{2}\sigma^{2}ab\left(1-q\right)x^{2}+\sigma x\right\}\times \\ & & {\left(1-\left(1-q\right)\beta \left(T\right)x^{2}\right)}^{\frac{1}{1-q}}dx+\int_{r_{2}}^{+\infty }\exp\left\{\frac{1}{2}\sigma^{2}ab\left(1-q\right)x^{2}+\sigma x\right\}\times \\ & & {\left(1-\left(1-q\right)\beta \left(T\right)x^{2}\right)}^{\frac{1}{1-q}}dx] \end{array}$$

◻

**Corollary** **1.** *The call-put parity relationship of geometric average Asian options is*

$$\begin{array}{cc} C_{q}\left(K,T\right)-P_{q}\left(K,T\right)= & \frac{S(0)}{z(T)}e^{-\frac{1}{2}rT-\frac{1}{2}\frac{3-q}{5-q}\sigma^{2}aT^{\frac{2}{3-q}}}\int_{-\infty }^{+\infty }\exp\{\frac{1}{2}\sigma^{2}ab\left(1-q\right)x^{2}\\ & +\;\sigma x\}\times {\left(1-\left(1-q\right)\beta \left(T\right)x^{2}\right)}^{\frac{1}{1-q}}dx-\frac{K}{z(T)}e^{-rT} \end{array}$$

In addition, under the framework of the Black-Scholes model, the underlying asset price is assumed to follow the geometric Brownian motion. That is, the underlying stock price S(t) satisfies the following stochastic differential equation

(29) dS(t)=μS(t)dt+σS(t)dW(t)

where W(t) is a standard Brownian motion defined on a complete probability space (Ω,F,P). Let $\tilde{W}\left(t\right)=W\left(t\right)+\int_{0}^{t}\theta ds$ and $\theta =\frac{\mu -r}{\sigma }$. Then, there is an equivalent martingale measure Q, which is related to P through the Radon-Nikodym derivative

$$\frac{dQ}{dP}=\exp\left(\int_{0}^{t}\theta dW\left(s\right)-\frac{1}{2}\int_{0}^{t}\theta^{2}ds\right)$$

According to the Girsanov theorem, we know that $\tilde{W}\left(t\right)$ is a standard Brownian motion under the probability measure Q. Using the method of lemma 2, we can obtain that the discounted stock price process $\tilde{S}\left(t\right)=S\left(t\right)e^{-rt}$ is a martingale and satisfies the following stochastic differential equation

$$d\tilde{S}\left(t\right)=\sigma \tilde{S}\left(t\right)d\tilde{W}\left(t\right)$$

By the martingale method, it is not difficult to derive the pricing formula of a geometric average Asian call option (29) in the framework of the Black-Scholes model. It satisfies the following equation

$$\begin{array}{ccc} C(K,T) & = & S\left(0\right)e^{-\frac{1}{2}T\left(r+\frac{1}{6}\sigma^{2}\right)}N\left(d_{1}\right)-Ke^{-rT}N\left(d_{2}\right) \end{array}$$

where $d_{1}=\frac{\ln S(0)-\ln K+\frac{1}{2}T\left(r+\frac{1}{6}\sigma^{2}\right)}{\sigma \sqrt{\frac{T}{3}}}$, $d_{2}=d_{1}-\sigma \sqrt{\frac{T}{3}}$, and the pricing formula of a geometric average Asian put option can be written as

$$\begin{array}{ccc} P(K,T) & = & Ke^{-rT}N\left(-d_{2}\right)-S\left(0\right)e^{-\frac{1}{2}T\left(r+\frac{1}{6}\sigma^{2}\right)}N\left(-d_{1}\right) \end{array}$$

## 4. Numerical Results

In this section we carry out a comparative analysis between our model and the Black-Scholes model based on the geometric Brownian motion. We use S(0)=50, T=0.5, r=0.5, σ=0.25, q=1.5. Without loss of generality, We can take μ=0. Then we use (2) to generate a stock price sample of size =5000. By fitting the geometric Brownian motion with μ=0, we obtain the parameter σ=0.29 for the Black-Scholes model.

**Figure 1:**
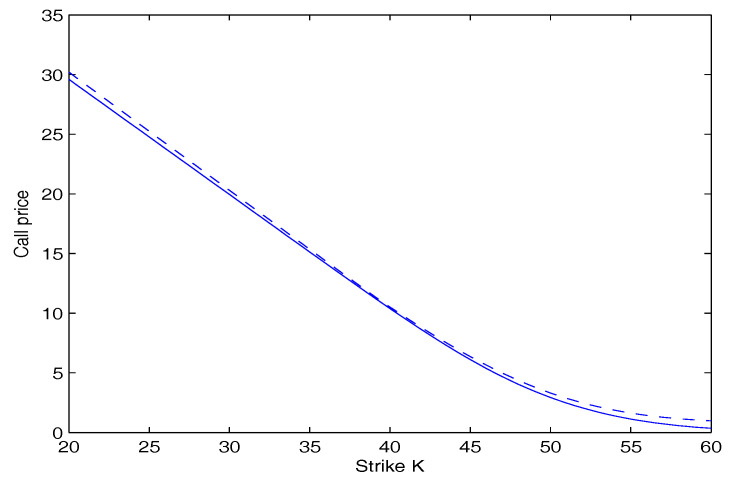
Call option price versus strike price. The dashed curve is for the Black-Scholes model. The solid curve is for our model.

**Figure 2:**
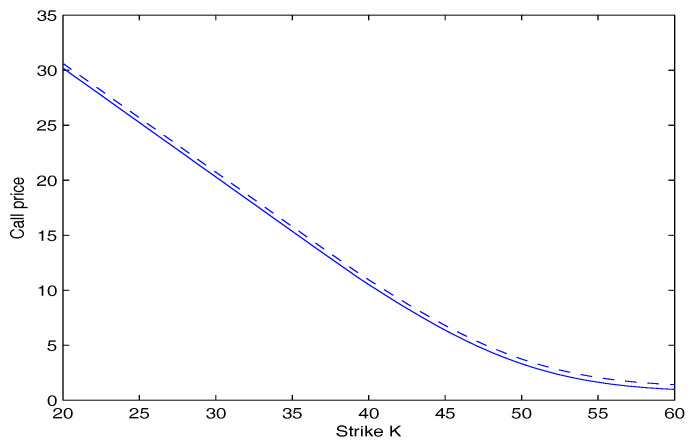
Put option price versus strike price. The dashed curve is for the Black-Scholes model. The solid curve is for our model.

In Figure 1, the call option price as a function of the strike price *K* is plotted. In Figure 2, the put option price as a function of the strike price *K* is plotted. These two figures show that the option price calculated by our model is lower than that calculated by the Black-Scholes model. Furthermore, the deviation is more significant as the strike price *K* goes away from the initial stock price S(0). This suggests that investors underestimate the risk using the Black-Scholes model.

**Figure 3:**
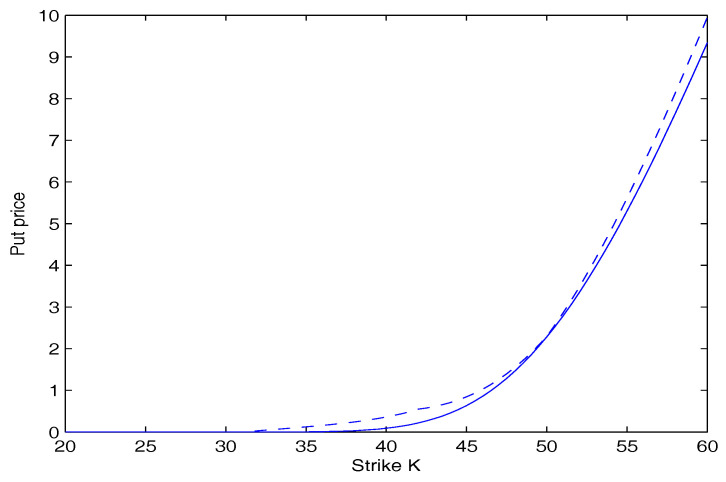
Call option price versus strike price, for different values of *q*. The dashed curve is for q=1.5. The Solid Curve is for q=1.4.

**Figure 4:**
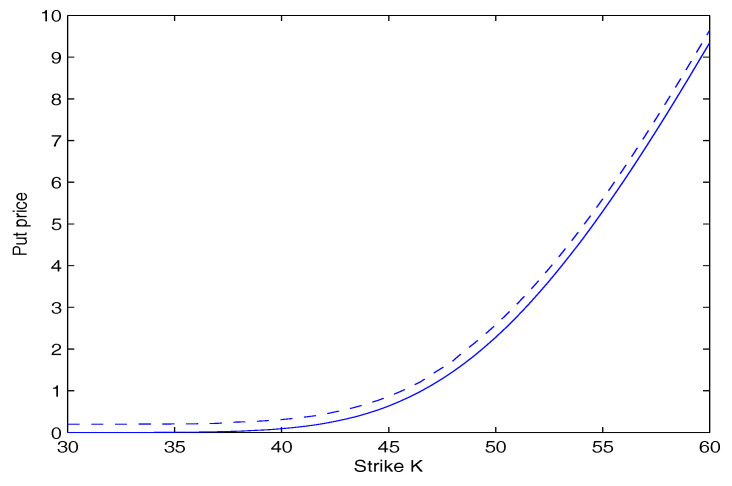
Put option price versus strike price, for different values of *q*. The dashed curve is for q=1.5. The Solid Curve is for q=1.4.

Figure 3 and Figure 4 depict the price of call and put option as a function of the strike price *K* for our model with q=1.5 and q=1.4 respectively. They show that under the same conditions, the price of the option increases with an increase of the value of *q*. This is because when the value of *q* increases, the tail of returns distribution is fatter.

## 5. Sumary

We know that it is very important to accurately model the price process of underlying assets, which is the foundation of derivatives pricing. In order to exactly describe asset price process, in this paper we employ a non-Gaussian stochastic process based on the non-extensive statistical mechanics framework, which can describe characteristics of long-run dependence of asset prices quite well. Moreover, by the use of martingale method, we obtain closed form solutions for geometric average Asian options.

As future work we will study the pricing of other exotic options and empirical analysis of the financial market under the non-extensive statistical mechanics framework.
